# Glioma Cells with the IDH1 Mutation Modulate Metabolic Fractional Flux through Pyruvate Carboxylase

**DOI:** 10.1371/journal.pone.0108289

**Published:** 2014-09-22

**Authors:** Jose L. Izquierdo-Garcia, Larry M. Cai, Myriam M. Chaumeil, Pia Eriksson, Aaron E. Robinson, Russell O. Pieper, Joanna J. Phillips, Sabrina M. Ronen

**Affiliations:** 1 Department of Radiology and Biomedical Imaging, University of California San Francisco, San Francisco, California, United States of America; 2 Department of Neurological Surgery, University of California San Francisco, San Francisco, California, United States of America; Albert Einstein College of Medicine, United States of America

## Abstract

**Background:**

Over 70% of low-grade gliomas carry a heterozygous R132H mutation in the gene coding for isocitrate dehydrogenase 1 (IDH1). This confers the enzyme with the novel ability to convert α-ketoglutarate to 2-hydroxyglutarate, ultimately leading to tumorigenesis. The major source of 2-hydroxyglutarate production is glutamine, which, in cancer, is also a source for tricarboxylic acid cycle (TCA) anaplerosis. An alternate source of anaplerosis is pyruvate flux via pyruvate carboxylase (PC), which is a common pathway in normal astrocytes. The goal of this study was to determine whether PC serves as a source of TCA anaplerosis in IDH1 mutant cells wherein glutamine is used for 2-hydroxyglutarate production.

**Methods:**

Immortalized normal human astrocytes engineered to express heterozygous mutant IDH1 or wild-type IDH1 were investigated. Flux of pyruvate via PC and via pyruvate dehydrogenase (PDH) was determined by using magnetic resonance spectroscopy to probe the labeling of [2-^13^C]glucose-derived ^13^C-labeled glutamate and glutamine. Activity assays, RT-PCR and western blotting were used to probe the expression and activity of relevant enzymes. The Cancer Genome Atlas (TCGA) data was analyzed to assess the expression of enzymes in human glioma samples.

**Results:**

Compared to wild-type cells, mutant IDH1 cells significantly increased fractional flux through PC. This was associated with a significant increase in PC activity and expression. Concurrently, PDH activity significantly decreased, likely mediated by significantly increased inhibitory PDH phosphorylation by PDH kinase 3. Consistent with the observation in cells, analysis of TCGA data indicated a significant increase in PC expression in mutant IDH-expressing human glioma samples compared to wild-type IDH.

**Conclusions:**

Our findings suggest that changes in PC and PDH may be an important part of cellular adaptation to the IDH1 mutation and may serve as potential therapeutic targets.

## Introduction

Gliomas are the most common primary malignancy of the brain. They have an annual incidence of 17,000 and are associated with a poor prognosis and quality of life [1]. Traditionally, histology has been used to distinguish between different types of glioma, and most notably between low-grade gliomas and high-grade glioblastoma, which is the most malignant subtype of glioma [2]. However, recent studies show that primary glioblastomas are genetically distinct from low-grade gliomas and their upgraded counterparts, secondary glioblastomas [1], [3]. Specifically, 60–90% of low-grade gliomas and secondary glioblastomas harbor a heterozygous R132H mutation in the gene coding for the cytosolic isoform of isocitrate dehydrogenase (IDH1) [4]. In contrast, only 6% of primary glioblastomas harbor this mutation [5]. The wild-type form of IDH1 catalyzes the conversion of isocitrate to α-ketoglutarate (α-KG) whereas the mutant enzyme acquires the novel ability to convert α-KG into 2-hydroxyglutarate (2-HG), a so-called “oncometabolite” not normally found in cells at appreciable concentrations [6]. When 2-HG is present at elevated levels, it has been shown to mediate the process of oncogenesis through competitive inhibition of multiple α-KG-dependent enzymes and subsequent alterations of the cellular epigenome [7].

Metabolically, glutamine is the primary source of 2-HG [8] and mutant IDH1 cells are uniquely sensitive to inhibition of glutaminase, the enzyme that catalyzes the conversion of glutamine to glutamate, which, in turn, can be converted to α-KG by transamination [9]. However, in proliferating cancer cells, exogenous glutamine is also known to play an important role in supporting cellular growth and proliferation [10]–[15]. Specifically, glutamine metabolism via glutaminolysis into glutamate, α-KG and the tricarboxylic acid (TCA) cycle serves to replenish TCA cycle intermediates lost in the production of cellular building blocks such as amino acids and fatty acids [12], [14], [15]. An alternate source for TCA cycle anaplerosis in tumor tissue is pyruvate flux via pyruvate carboxylase (PC), generating oxaloacetate. PC is considered the archetypical anaplerotic enzyme with high activities in many human tissues [16]. In normal astrocytes, PC has been shown to play an essential role in the synthesis of TCA cycle-derived neurotransmitters, including glutamate, GABA, and aspartate [17]–[20]. In addition, up-regulation of PC has been shown to enable glutamine-independent growth of wild-type IDH1 glioblastoma cells [21], indicating that PC can serve as a metabolic alternative to glutamine.

In this context, we questioned whether PC could provide a significant source of TCA anaplerotic flux in mutant IDH1 glioma cells wherein significant amounts of glutamine are channeled towards 2-HG production. To address this question, we used magnetic resonance spectroscopy (MRS) to explore the metabolism of [2-^13^C]glucose into ^13^C-labeled glutamate and ^13^C-labeled glutamine. By probing the ^13^C-labeling pattern of these metabolites, we were able to quantify the fractional flux of glucose-derived pyruvate via PC and pyruvate dehydrogenase (PDH), a non-anaplerotic pathway supporting the complete oxidation of pyruvate [22]. We show that fractional PC flux was elevated in cells expressing mutant IDH1 when compared to cells expressing wild-type IDH1. We also demonstrate that this effect was likely mediated by an increase in the expression and cellular activity of PC as well as a drop in cellular activity of PDH. Importantly, analysis of The Cancer Genome Atlas (TCGA) data demonstrates that the expression of PC was also significantly elevated in mutant IDH-expressing tumors when compared to wild-type IDH tumors, highlighting the clinical significance of our observations.

## Materials and Methods

### Cell culture

Immortalized normal human astrocytes E6/E7/hTERT (NHA) expressing either the wild-type IDH1 gene (IDHwt cells) or the heterozygous R132H mutant IDH1 gene (IDHmut cells) were generated in the Pieper laboratory [23] by lentiviral transduction as previously described [24]. Cell lines were regularly authenticated by analysis of short tandem repeats (UCSF Genome Core).

Cell cultures were routinely maintained in high-glucose (4.5 g/L) Dulbecco’s modified Eagle’s medium (DMEM; UCSF Cell Culture Facility), supplemented with 876 mg/L glutamine (Invitrogen), 10% heat-inactivated fetal bovine serum (Thermo Scientific Hyclone), 100units/mL penicillin and 100 ug/mL streptomycin (UCSF Cell Culture Facility) at 37°C in 5% CO_2_. All experiments were performed when cells were in the logarithmic growth phase.

### Metabolic flux analysis

2×10^7^ NHA cells were grown for 18 hours in custom-made glucose-free DMEM (UCSF Cell Culture Facility) supplemented with 1 g/L [2-^13^C]glucose (Sigma). After 18 hours, we confirmed by ^13^C-MRS that glucose was not depleted from the medium. Cells were then extracted using the dual-phase extraction method as previously described [25], [26]. Briefly, cells were washed with saline to remove residual medium and fixed with 10 mL each of ice-cold methanol, chloroform, and deionized water. Following phase separation, the aqueous phase was lyophilized and resuspended in 400 µL of deuterium oxide (Cambridge Isotope Laboratories)-based potassium phosphate buffer at pH = 7.

Extracts were analyzed using a 500 MHz Bruker Avance spectrometer equipped with a Triple Resonance CryoProbe. Proton-decoupled ^13^C spectra were obtained using a 30^o^ flip angle and 3 second relaxation delay averaged over 2048 acquisitions. In addition a fully relaxed spectrum (90° pulse, relaxation delay of 60 s and broad-band decoupling applied during the acquisition time) was recorded and served to determine the saturation and Nuclear Overhauser effect (NOE) correction factors. All spectral assignments were based on literature reports and www.hmdb.ca. Data analysis was performed using MestRenova software (Mestrelab). Quantification of the peaks was achieved by measuring peak integrals after deconvolution of overlapping peaks, normalizing to cell number and to an external reference of known concentration (1 mM [1-^13^C]glucose) and correction for saturation and NOE.

**Figure 1:**
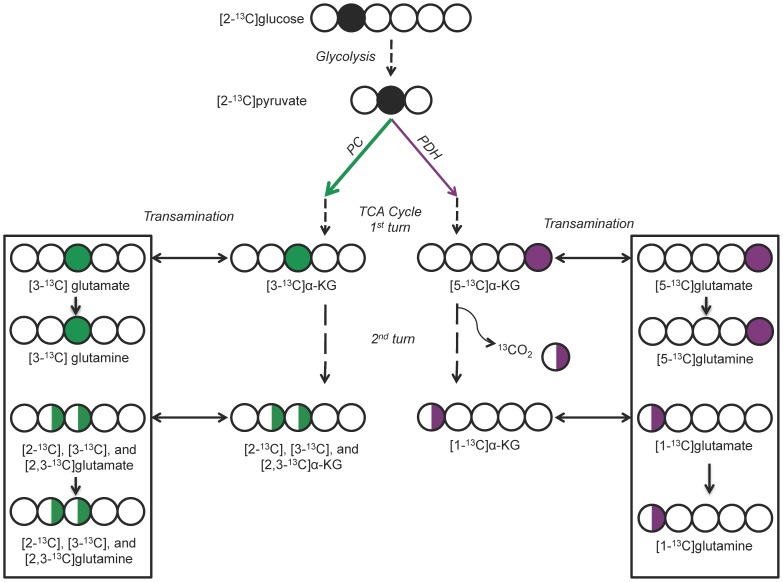
^13^C labeling patterns of glutamate derived from [2-^13^C]glucose. Plain arrows represent flux through the indicated enzymatic process. Dotted arrows represent flux through the indicated multistep metabolic pathway. Circles represent the carbon backbone of the molecule. Filled black circles indicate the location of the ^13^C label upstream of PC and PDH. Filled green and purple circles indicate the location of the ^13^C label after metabolism through PC or PDH, respectively, and the first turn of the TCA cycle. Half-filled green and purple circles indicate the locations of the ^13^C label after metabolism through the second and subsequent turns of the TCA cycle on a population level (i.e.: may or may not be on the same molecule). The boxes represent the glutamate/glutamine molecules detected in the MR spectra. Adapted from Brekke et al. [27]. PC = pyruvate carboxylase, PDH = pyruvate dehydrogenase, TCA = tricarboxylic acid, α-KG = α-ketoglutarate.



To determine the fractional flux of glucose-derived pyruvate via PC or PDH, we used the approach previously described by Brekke *et al*. [27]. As previously shown, [2-^13^C]glucose is metabolized via glycolysis to pyruvate, and pyruvate enters the TCA cycle through either PC or PDH. It is then converted to α-KG, which in turn is converted to glutamate and then to glutamine. [2-^13^C]glucose leads to ^13^C labeling of glutamate carbons that specifically reflect flux via PC or PDH. As a result, ^13^C MRS of labeled glutamate/glutamine can be used to distinguish between pyruvate flux through PC or PDH. As illustrated in Figure 1, during the first turn of the TCA cycle after the PC or PDH reaction, the ^13^C label produces [3-^13^C]glutamate/glutamine or [5-^13^C]glutamate/glutamine, respectively. The subsequent turn of the TCA cycle leads to additional labeling of [2-^13^C]glutamate/glutamine for PC and [1-^13^C]glutamate/glutamine and ^13^CO_2_ for PDH. Detailed analysis (including backflux from oxaloacetate to succinate [27]) indicates that the fractional flux of PC and PDH is proportional to the MRS intensities of the respective labeled glutamate carbons, as expressed in the following equations [27]:

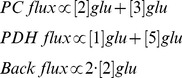



Thus, fractional flux is equal to:



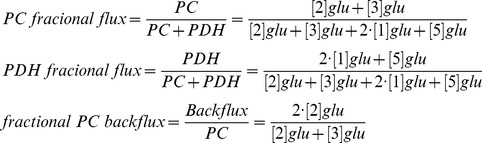
where [#]Glu represents the MRS intensity of the indicated ^13^C-labeled glutamate carbon. Similar equations are valid for glutamine signals.

### QRT-PCR Gene expression analysis

RNA was isolated from 5×10^6^ cells using Rneasy kit (Qiagen) using manufacturer’s instructions, and analyzed as follows at the Genome Analysis Core Facility, Helen Diller Family Comprehensive Cancer Center at the University of California, San Francisco. cDNA was generated from RNA samples using the qScript cDNA Synthesis Kit (Quanta Biosciences) using manufacturer’s instructions on a PTC-225 Thermocycler (MJ Research). Quantitative, real-time polymerase chain reaction was conducted with 20 µL reaction volumes of 1X Taqman buffer (Applied Biosystems), 5.5 mM MgCl_2_, 0.5 mM of each primer and 200 nM of the corresponding Taqman probe (Life Technologies), 0.2 µM of each deoxynucleotide triphosphate, 0.025unit/µL AmpliTaq Gold (Applied Biosystems), and 5 ng cDNA in a 384-well plate. The ABI 7900HT (Applied Biosystems) instrument was used with the following parameters: 1 cycle of 95°C for 10 minutes and 40 cycles of 95°C for 15 seconds, 60°C for 1 minute. Analysis was carried out using SDS software (version 2.3) to determine Ct. Expression levels of transcripts were normalized to the glyceraldehyde-3-phosphate dehydrogenase (GAPDH) transcript.

### Western blot analysis

Protein was isolated from 1×10^7^ cells using lysis buffer (Cell Signaling) in the presence of protease inhibitor (Calbiochem). The Bradford method was used to calculate protein concentrations in order to load equivalent amounts of protein between samples. The protein-containing lysate was subjected to polyacrylamide gel electrophoresis (BioRad) under denaturing conditions, followed by transfer to PVDF membrane (Millipore). Membranes were blocked using 5% milk (Santa Cruz Biotechnology) and primary antibodies (Abcam: PC and PDH) (Cell Signaling: GAPDH and Tubulin) were incubated overnight at 4°C in 5% BSA (Sigma). HRP-conjugated secondary antibodies (Cell Signaling) were incubated for 90 mins in 5% milk at room temperature. Enhanced chemiluminescence substrate (Thermo Scientific) was used to develop the membrane onto film. Densitometry of bands was performed using ImageJ software (NIH) to quantify protein expression. Data was normalized to protein levels of Tubulin or GAPDH.

### Enzyme activity assays

PDH activity was determined using a commercially available assay kit (Abcam) and performed according to manufacturer’s instructions. 1×10^7^cells were lysed using the provided lysis buffer supplemented with 20 mM sodium fluoride (Sigma), a phosphatase inhibitor, 4units/mL apyrase (Sigma), an ATP depletor, and a protease inhibitor cocktail (Calbiochem) to preserve protein integrity and phosphorylation status. Lysates were incubated in the Abcam-supplied microplate and reaction mix. The PDH reaction was measured spectrophotometrically in kinetic mode over 30 minutes at 450 nm (Tecan) and normalized to cell number.

PC activity assay was adapted from a protocol developed by the Comprehensive Molecular BioEngineering department at the University of Georgia in which cellular PC converts exogenous pyruvate and bicarbonate to oxaloacetate, then exogenous citrate synthase converts oxaloacetate and exogenous acetyl-CoA into citrate and free Coenzyme A (CoA). The free thiol group on the CoA molecule then reduces exogenous Ellman’s reagent to a product detectable at 412 nm. Here 1×10^6^ cells were lysed with lysis buffer (Cell Signaling) in the presence of protease inhibitor (Calbiochem). 35 µL of lysate was added to 465 µL of assay buffer containing 95 mM Tris-HCl (pH = 8), 53 mM sodium bicarbonate, 5.3 mM magnesium chloride, 53 mM ATP, 53 mM sodium pyruvate, 0.11 mM acetyl-CoA, 0.1 mg/mL Ellman’s reagent, and 4units/mL citrate synthase (all reagents from Sigma). A spectrophotometric reading in kinetic mode at 412 nm was taken for 10 minutes at 30°C (Tecan). Background readings were obtained from samples in which 40 µg/mL avidin (Sigma), an inhibitor of pyruvate carboxylase, was added. Data was corrected for background and normalized to cell number.

### PDH phosphorylation assay

PDH phosphorylation was determined using an ELISA kit (Abcam) that was run according to manufacturer’s instructions. Cells grown in a 96-well plate were fixed with 4% paraformaldehyde then incubated in phospho-PDH antibodies (Ser293, Ser300, or Ser232) and PDH E1 subunit antibody at 4°C. Following this, cells were incubated in two secondary antibodies: one against the phospho-PDH antibody and conjugated to horseradish peroxidase, and another against the PDH E1 subunit antibody and conjugated to alkaline phosphatase. An alkaline phosphatase substrate was then added and color development was measured spectrophotometrically in kinetic mode at 405 nm (Tecan); the rate of color development represents the quantity of total PDH. A horseradish peroxidase substrate was subsequently added and color development was measured spectrophotometrically in kinetic mode at 600 nm (Tecan); the rate of color development represents the quantity of phospho-PDH at Ser232, Ser293, or Ser300. Whole cell staining with Janus Green was also performed for 10 minutes and measured at 595 nm (Tecan) to determine cell number. Data was normalized to total PDH and cell number.

### Statistical analysis of cell data

All cell study results are expressed as mean±SD, representing the results of at least three repeats. Statistical significance was determined using a two-tailed, unpaired Student's t-test assuming unequal variance, with p<0.05 considered to be significant.

### Analysis of The Cancer Genome Atlas data

Data from The Cancer Genome Atlas (TCGA) Data Portal (http://cancergenome.nih.gov.) were downloaded from the CBio Portal for Cancer Genomics (http://www.cbioportal.org/public-portal/index.do) and mean normalized expression scores (z-scores) for enzymes of interest determined. Statistical significance of differences was determined using a two-tailed Mann Whitney test, with p<0.05 considered to be significant.

## Results

**Figure 2:**
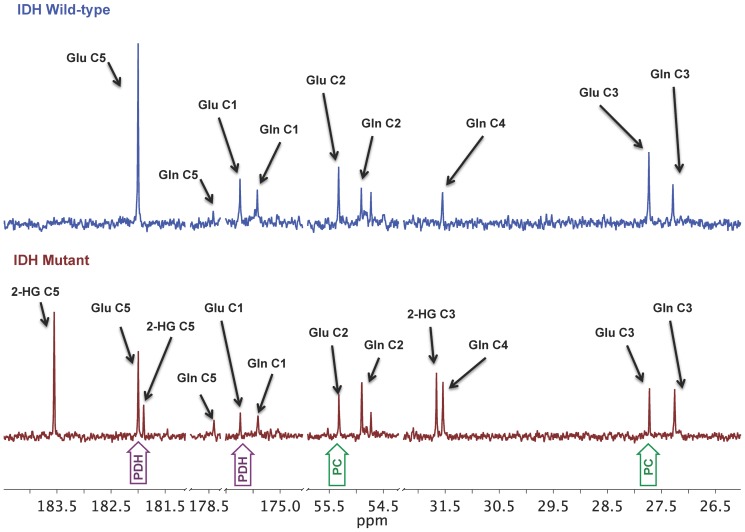
Representative ^13^C MR spectra of cell extracts post-incubation with [2-^13^C]glucose. MR spectroscopy was performed on the aqueous phase of NHA IDH1 wild-type (top) and IDH1 mutant (bottom) cell extracts following 18 hours of incubation with medium containing 1 g/L [2-^13^C]glucose. [^13^C]glutamate peaks relevant for calculating pyruvate carboxylase and pyruvate dehydrogenase fractional fluxes, namely [1-^13^C], [2-^13^C], [3-^13^C], and [5-^13^C]glutamate, are highlighted in green (flux via pyruvate carboxylase) and purple (flux via pyruvate dehydrogenase). This data combined with 4 other spectra served to generate the results presented in Figure 3 and Table 1. Glu = glutamate, Gln = glutamine, 2-HG = 2-Hydroxyglutarate, IDH = isocitrate dehydrogenase, PC = pyruvate carboxylase, PDH = pyruvate dehydrogenase.

**Figure 3:**
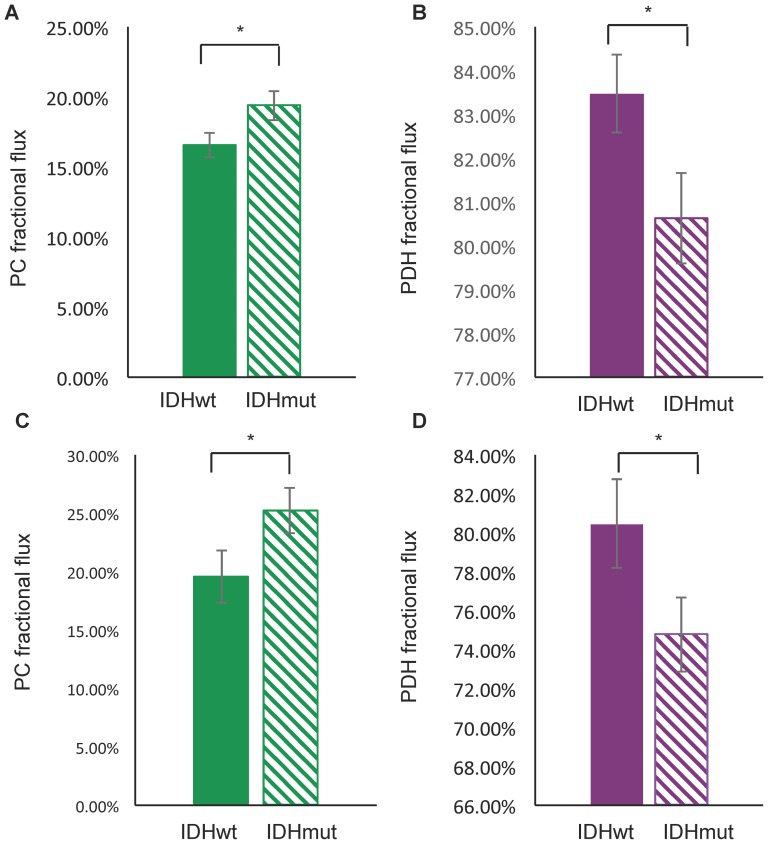
[2-^13^C]glucose-derived fractional flux to glutamate and glutamine. Fractional flux to glutamate (A&B) and to glutamine (C&D) via pyruvate carboxylase (in green) and (pyruvate dehydrogenase (in purple) for NHA IDHwt (solid) and IDHmut (striped) cells (data presented are averages of 3 repeats per cell line). Error bars represent standard deviations. Asterisks represent statistical significance (*: p<0.05). PC = pyruvate carboxylase, PDH = pyruvate dehydrogenase, IDHwt = isocitrate dehydrogenase wild-type, IDHmut = isocitrate dehydrogenase mutant.

The ^13^C-labeling pattern of glutamate and/or glutamine generated from the metabolism of [2-^13^C]glucose can be used to estimate the fractional flux of glucose-derived pyruvate via PC or PDH (Figure 1) [27]. To determine whether fractional flux via PC was increased in mutant IDH1 cells compared to wild-type, we therefore incubated cells with [2-^13^C]glucose and acquired ^13^C MR spectra of cell extracts post incubation (Figure 2). Prominent metabolites derived from [2-^13^C]glucose included 2-HG in IDHmut cells, and taurine, alanine, glutamine, and glutamate in both IDHmut and IDHwt cell lines. [3-^13^C]glutamate (27.6 ppm) and [3-^13^C]glutamine (27.1 ppm) observed in our spectra reflect the first turn of the TCA cycle after pyruvate flux through PC whereas [2-^13^C]glutamate (55.4 ppm) and [2-^13^C]glutamine (54.9 ppm) result from the second turn. [5-^13^C]glutamate (182.0 ppm) and [5-^13^C]glutamine (178.4 ppm) result from the first turn of the TCA cycle after pyruvate flux through PDH, and [1-^13^C]glutamate (175.6 ppm) and [1-^13^C]glutamine (175.2 ppm) result from the second turn of the TCA cycle. When comparing mutant and wild-type IDH1 cells, we observed a significant decrease in both the PDH-derived and PC-derived labeled glutamate levels (Table 1). Specifically, PDH-derived [5-^13^C] glutamate plus [1-^13^C] glutamate decreased 42% from 2.26±0.29 to 1.31±0.21 fmol/cell (p<0.05, n = 3) and PC-derived [2-^13^C] glutamate plus [3-^13^C] glutamate decreased 26% from 0.46±0.07 to 0.34±0.06 fmol/cell (p<0.05, n = 3) (Table 1). However, when considering the PC and PDH fractional fluxes calculated from the labeled glutamate signals (Table 1 & Figure 3), we found that PC fractional flux increased significantly from 17%±2% in IDHwt to 19%±2% in IDHmut cells (Figure 3A, p<0.01, n = 3). PDH fractional flux decreased from 83%±2% in IDHwt to 81%±2% (Figure 3B, p<0.05, n = 3). Our results were similar when calculating the PC and PDH fractional fluxes based on ^13^C glutamine signals (Table 1 & Figure 3). PC flux increased from 20%±5% in IDHwt to 25%±4% in IDHmut (Figure 3C, p<0.05, n = 3) and PDH flux dropped from 80%±5% in IDHwt to 75%±4% (Figure 3D, p<0.05, n = 3).

**Table 1:** ^13^C labeling of glutamate and glutamine (fmol/cell and nmol/mg protein) from [2-^13^C]glucose, and PC, PDH and backflux fractional fluxes in IDHwt and IDHmut immortalized normal human astrocytes.

	IDHwt	IDHmut	IDHwt	IDHmut
Glutamate	Concentration (fmol/cell)		Concentration (nmol/mg protein)	
[5-^13^C] Glutamate	1.76±0.10	1.02±0.16*	1.23±0.07	0.75±0.12*
[1-^13^C] Glutamate	0.50±0.20	0.28±0.05	0.35±0.14	0.21±0.04
[2-^13^C] Glutamate	0.27±0.03	0.16±0.05*	0.19±0.02	0.12±0.03*
[3-^13^C] Glutamate	0.28±0.06	0.22±0.01	0.19±0.04	0.16±0.01
[5-^13^C] Glutamate + [1-^13^C] Glutamate	2.26±0.29	1.31±0.21*	1.58±0.20	0.95±0.16*
[2-^13^C] Glutamate + [3-^13^C] Glutamate	0.46±0.07	0.34±0.06*	0.32±0.05	0.25±0.04*
	Fractional Flux (%)			
PC/(PDH+PC)	17±2	19±2*		
PDH/(PDH+PC)	83±2	81±2*		
Backflux/PC	99±12	84±11		
Glutamine	Concentration (fmol/cell)		Concentration (nmol/mg protein)	
[5-^13^C] Glutamine	0.26±0.05	0.29±0.20	0.18±0.03	0.20±0.14
[1-^13^C] Glutamine	0.79±0.29	0.80±0.49	0.55±0.20	0.56±0.34
[2-^13^C] Glutamine	0.17±0.01	0.24±0.08	0.12±0.01	0.17±0.06
[3-^13^C] Glutamine	0.27±0.04	0.34±0.18	0.19±0.03	0.24±0.13
[5-^13^C] Glutamine + [1-^13^C] Glutamine	1.04±0.09	1.09±0.53	0.73±0.06	0.76±0.37
[2-^13^C] Glutamine + [3-^13^C] Glutamine	0.45±0.03	0.58±0.15	0.31±0.02	0.41±0.10
	Fractional Flux (%)			
PC/(PDH+PC)	20±5	25±4*		
PDH/(PDH+PC)	80±5	75±4*		
Backflux/PC	77±7	85±7		

IDHwt (n = 3) and IDHmut (n = 3) NHA cell extracts were analyzed by MR spectroscopy following 18 hours of incubation with medium containing 1 g/L [2-^13^C]glucose. Fractional fluxes were calculated based on the intensities of the four labeled peaks. Asterisks represent statistical significance (*: p<0.05). PC = pyruvate carboxylase, PDH = pyruvate dehydrogenase, IDHwt = isocitrate dehydrogenase wild-type cells, IDHmut = isocitrate dehydrogenase mutant cells.

As previously described [27], the above fractional fluxes also include backflux of PC-derived oxaloacetate, which will generate equal amounts of [2-^13^C] and [3-^13^C] fumarate. When followed by forward flux and condensation with acetyl CoA, these give rise to equal amounts of [3-^13^C] and [4-^13^C] citrate, and thereby equal amounts of [2-^13^C] and [3-^13^C] glutamate. Based on our spectra we were therefore also able to determine the backflux-to-PC ratio and found that it represents 99±12% of the PC flux in IDHwt cells and 84±11% in IDHmut cells. The difference between IDHwt and IDHmut cells was not significant (Table 1).

**Figure 4:**
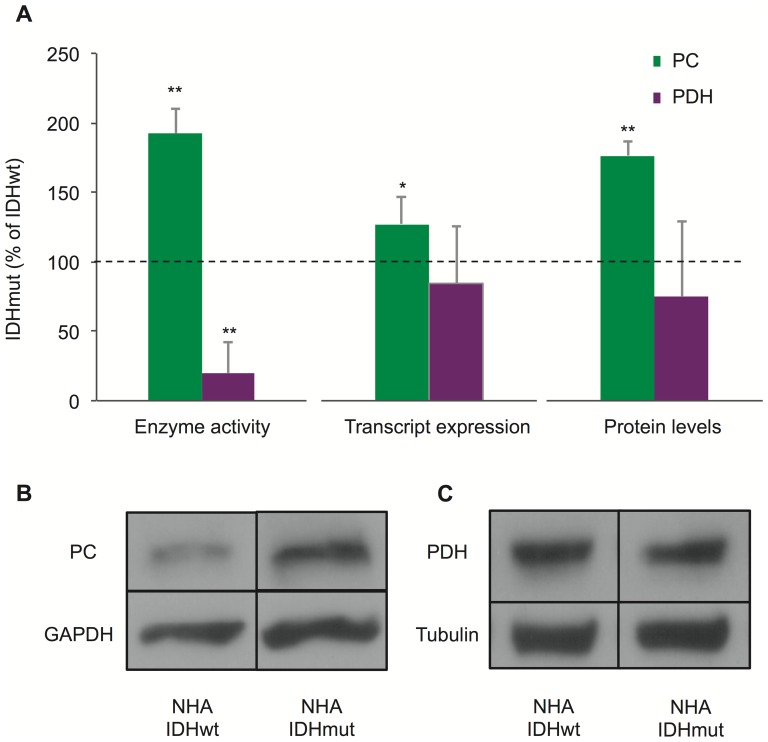
Pyruvate carboxylase and pyruvate dehydrogenase enzyme activities, expression and protein levels. (**A**) Enzymatic activities (n = 3), transcript levels (n = 9), and protein levels (n = 3) for pyruvate carboxylase (green) and pyruvate dehydrogenase (purple) in NHA IDHmut cells expressed as a percentage of their IDHwt counterparts. The horizontal dotted lines represents the baseline (i.e.: 100% of IDHwt). Error bars represent standard deviation. Asterisks represent statistical significance (*: p<0.05, **: p<0.01). (**B**) Representative Western blot bands used to quantify pyruvate carboxylase protein levels of NHA IDHwt and IDHmut cells. (**C**) Representative Western blot bands used to quantify pyruvate dehydrogenase protein levels of NHA IDHwt and IDHmut cells. Pyruvate carboxylase and pyruvate dehydrogenase quantification were normalized to glyceraldehyde-3-phosphate dehydrogenase and tubulin respectively. PC = pyruvate carboxylase, PDH = pyruvate dehydrogenase, IDHwt = isocitrate dehydrogenase wild-type, IDHmut = isocitrate dehydrogenase mutant, GAPDH = glyceraldehyde-3-phosphate dehydrogenase.

The changes in fractional flux of PC and PDH could be caused by an increase in cellular PC activity, a decrease in cellular PDH activity, or both. To address this point, we measured the cellular enzyme activities of PC and PDH in IDHwt and IDHmut cells using spectrophotometric assays (Figure 4A). Consistent with the changes in fractional flux, we found that PC activity increased significantly by 92%±18% (p<0.01, n = 3) in IDH1mut cells compare to IDH1wt. In addition, cellular PDH activity decreased by 80%±22% (p<0.01, n = 3) in the IDHmut cells. Thus both an increase in cellular PC activity and a decrease in cellular PDH activity likely contribute to the change in fractional fluxes observed in our NHA cells expressing mutant IDH1 when compared to cells expressing wild-type IDH1.

In an effort to explain the changes in cellular enzyme activities, we investigated whether the transcript expression of PC and PDH was altered in cells expressing mutant IDH1 (Figure 4B). Consistent with the activity findings, PC transcript expression increased significantly by 27%±19% in IDHmut compared to IDHwt cells (p<0.05, n = 9 Figure 4B). However, no significant changes in PDH transcript levels were detected. Protein levels, as revealed by Western blot, confirmed these observations (Figures 4C, D and E). PC protein levels increased significantly by 76%±10% in NHA IDHmut compared to IDHwt (p<0.01, n = 3) (Figure 4D); however, no significant differences were detected in protein levels for PDH (Figure 4E). Thus, enzyme expression may explain the increase in PC activity in IDHmut cells, but this is unlikely the case for PDH.

The activity of PDH is also modulated by inhibitory phosphorylation at three serine sites: Ser232, Ser293, and Ser300. To assess whether phosphorylation could provide an explanation for our findings, we determined the levels of phospho-Ser232, 293, and 300 (Table 1). Compared to their IDHwt counterparts, IDHmut cell line had significantly higher levels of phospho-Ser293 and 300: phospho-Ser293 levels increased by 55%±30% (p<0.05, n = 3) and phospho-Ser300 levels increased by 52%±35% (p<0.05, n = 3). No significant differences were detected in Ser232.

Four isoforms of PDKs and two isoforms of PDH phosphatases (PDPs) control the levels of PDH phosphorylation [28]. QRT-PCR was therefore performed to probe the expression of these PDKs and PDPs in IDHwt and IDHmut cells (Table 2). Both PDK1 and PDK3 showed significant, high magnitude increases in transcript expression in IDHmut cells compared to IDHwt. PDK1 and PDK3 transcript expressions increased by 47%±13% (p<0.01, n = 9) and 305%±12% (p<0.01, n = 9) respectively in NHA IDHmut cells. In addition, substantially smaller expression changes were also observed in PDP2, which increased by 21%±8% (p<0.05, n = 9).

**Table 2:** Inhibitory pyruvate dehydrogenase phosphorylation and pyruvate dehydrogenase kinases and phosphatases transcript expression.

Cell Line	Ser232 (%)	Ser293 (%)	Ser300 (%)	PDK1 (%)	PDK2 (%)	PDK3 (%)	PDK4 (%)	PDP1 (%)	PDP2 (%)
NHA	110±27	155±30*	152±35*	147±13**	109±11	405±12**	not detected	104±11	121±8*

Inhibitory phosphorylation levels at three pyruvate dehydrogenase serine residues and transcript expression of pyruvate dehydrogenase kinases and phosphatases for NHA IDHmut cells expressed as a percentage of their IDHwt counterparts. Results are expressed as mean±SD (n = 3 for inhibitory phosphorylation; n = 9 for transcript expression). Asterisks represent statistical significance (*: p<0.05, **: p<0.01). Ser = serine, PDK = pyruvate dehydrogenase kinase, PDP = pyruvate dehydrogenase phosphatase, NHA = normal human astrocytes.

**Figure 5:**
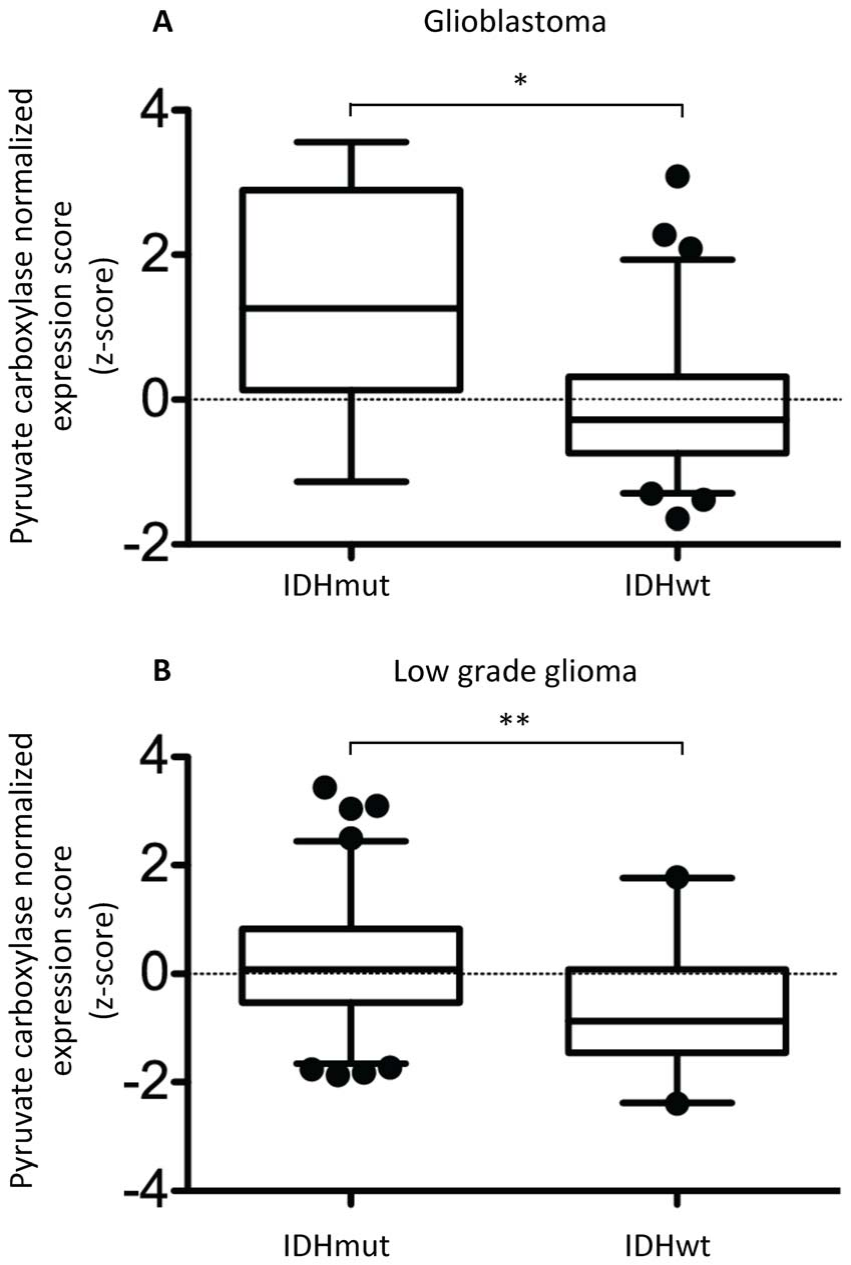
Comparison of normalized expression scores (z-scores) for pyruvate carboxylase across *de novo* glioblastoma (A) and low grade glioma (B). Boxes denote mean z-score and whiskers denote 2.5 to 97.5% for tumors in the specified group; IDH1/IDH2 mutant n = 8 and n = 174 and IDH wild-type n = 146 and n = 44 in (A) and (B), respectively. Data obtained from The Cancer Genome Atlas Data Portal (http://cancergenome.nih.gov.) A negative z-score denotes expression value below the glioblastoma population mean. Asterisks represent statistical significance (* p<0.01, ** p<0.0001). abbreviations.

To assess the clinical significance of our findings, we also analyzed human biopsy data reported by TCGA. As illustrated in Figure 5, the clinical findings with regard to PC were in line with our cell data. The mean normalized expression scores (z-scores) for PC were significantly elevated in IDH mutant *de novo* glioblastoma (1.364 vs. −0.1322, n = 8 and n = 146, respectively, p<0.0057). The elevation in PC expression was even more pronounced in lower grade gliomas for which the mean normalized expression scores for PC were 0.1842 in IDH mutant glioma (n = 174) as compared to −0.6931 in IDH wild-type glioma (n = 44, p<0.0001). The lower grade glioma results with regard to PDK expression were less consistent with the cells findings. PDK1 (IDHwt z-score: 0.3208, IDHmut z-score: −0.1288, p<0.0001) and PDK3 (IDHwt z-score: 0.7687, IDHmut z-score: −0.08545, p<0.0001) expression levels were down whereas PDK4 (IDHwt z-score: −0.4452, IDHmut z-score: 0.07543, p = 0.0004) expression level was increased (IDHwt n = 44, IDHmut n = 174).

## Discussion

Deregulated cellular metabolism is increasingly recognized as a hallmark of cancer [15], [29], [30]. Amongst a range of metabolic alterations, highly proliferative cancer cells often use glutamine as a source of TCA cycle anaplerosis, supporting the synthesis of TCA cycle-derived substrates, such as amino acids and fatty acids [10]–[12], [14], [15]. In mutant IDH1 glioma cells, glutamine is used for the production of 2-HG [8]. The goal of this study was to determine whether mutant IDH1 cells also increase their fractional flux via PC as a source of TCA anaplerosis.

By probing the metabolism of [2-^13^C]glucose into ^13^C-labeled glutamate in mutant and wild-type IDH1-expressing cells, we found that PC fractional flux ranges from 16% to 19% in our cells and that this flux increased significantly in mutant IDH1 cells when compared to wild-type cells. The fractional PC flux observed in our cells is within the wide 4% to 65% range previously reported a variety of *in vivo* [31] and cultured cell systems (calculated from [27]). However, it should be noted that whereas astrocytic oxidative metabolism is quite variable in cultured normal astrocytes [32]–[34], PC flux represents about 40% of the pyuruvate flux in normal brain [17], [31]. In contrast, previous studies in glioblastoma cells indicate that the pyruvate flux via PC is much lower. One previous study in SF188 cells reported no detectable pyruvate flux via PC [35] whereas another study reported that PC represents 14% of the total pyruvate flux in SFxl cells [36]. Thus glioblastoma cells generally appear to have a lower PC flux than the normal brain, but, as indicated in our study, mutant IDH1 cells have a higher PC flux that wild-type IDH1 cells. This would indicate that mutant IDH1-expressing cells reduce their PC flux during transformation to a somewhat lesser degree than wild-type IDH1 cells.

When specifically comparing our results to those observed in normal astrocytes [27] the level of backflux is within experimental error of values reported in normal astrocytes, which represent 79% to 83% of PC flux. Furthermore, PDH-derived glutamate labeling in our wild-type IDH1 cells was also comparable, within experimental error, to values reported by Brekke et al. [27] at 1.58 nmol/mg protein in our cells versus 1.24 nmol/mg protein in normal cerebral astrocytes. However, PC-derived labeling was notably lower in our cells at 0.46 nmol/mg protein compared to 2.31 nmol/mg protein in normal astrocytes and consistent with the above mentioned drop in PC flux when comparing tumor cells to normal brain.

When considering the level of ^13^C glutamate labeling in our mutant and wild-type IDH1 cells, it is of interest to compare this to the total cellular content of glutamate previously reported in our cells based on their ^1^H-MRS spectra [37]. Total glutamate levels were 7.80 fmol/cell in IDHwt cells and dropped to 4.50 fmol/cell in IDHmut cells. Accordingly labeling of the PDH-derived glutamate pools was comparable in our IDHwt and IDHmut cells, representing about 23% of the total cellular glutamate pool. This level of labeling is consistent with previously reported elevated levels of glutaminolysis in tumor cells, and with exogenous glutamine as an alternate source for glutamate [35].

In our analysis we assumed that labeling occurred via the first and second turns of the TCA cycle. A potential limitation of the current study may therefore be associated with glutamate and glutamine labeling via the third and subsequent turns of the TCA cycle [38], which cannot be ruled out. This would not affect the analysis of PDH-derived carbons in which subsequent turns of the TCA continue to label [1-^13^C]glutamate. However, flux via PC could lead to a small amount of [1-^13^C]glutamate/glutamine being labeled via the third turn of the TCA cycle. This would lead to an overestimate on our part of the PDH flux which leads to labeling of [1-^13^C]glutamate/glutamine via the second turn of the TCA cycle. However, when considering our results as a percentage of the total glutamate pool, we find that in wild-type cells PDH-derived glutamate labeled in the first turn ([5-^13^C]glutamate) represents 23% of the total glutamate pool, PDH-derived glutamate labeled in the second turn ([1-^13^C]glutamate) represents 6% of the total glutamate pool, and PC-derived glutamate labeled after the second turn ([2-^13^C]glutamate) represents 3% of the total glutamate pool. We therefore believe that the error in ignoring the third TCA turn of PC-derived labeling is relatively small and not likely to significantly affect our results. Furthermore, it is important to note that our MRS findings were validated by complementary assays and elevated levels of PC expression were also observed in TCGA clinical samples.

It has previously been shown that 2-HG can cause global changes in gene expression by altering the activity of JmjC histone 5-methylcytosine hydroxylases and TET cytosine demethylases, [7]. In particular, 2-HG can lead to changes in promoter methylation for some metabolic enzymes [39]. In our model the IDH1 mutation was accompanied by an increase in cellular PC activity, as well as an increase in PC expression. To our knowledge, no studies have reported on epigenetic changes associated with PC, and further studies are needed to assess the reasons for the increase in PC expression in mutant IDH1 cells. Nonetheless, our analysis of TCGA data indicates that PC expression is significantly up in mutant IDH-expressing human tumors when compared to wild-type IDH, both in the low-grade and high-grade cohorts, highlighting the clinical significance of our observations. Thus, PC flux could serve as a source of TCA anaplerosis in mutant IDH1 cells that channel glutamine to 2-HG production. As such, increased PC expression likely contributes to the metabolic adaptation of mutant IDH1 cells.

Our metabolic analysis in cells also allowed us to measure the fractional pyruvate flux through PDH, an enzyme that supports the complete oxidation of pyruvate carbons for aerobic ATP generation [22]. Our IDHmut cell line showed decreased fractional flux through PDH, accompanied by a decrease in cellular PDH activity, which is most likely due to the increase in inhibitory phosphorylation of PDH observed at Ser293 and 300. The level of phosphorylation can be controlled by four PDKs and two PDPs. In our model, small changes were observed in the expression of PDP2 (increase) that would lead to an increase rather than a drop in PDH activity. In contrast, very substantial and significant increases were observed in PDK1 and PDK3 transcripts. Both kinases can phosphorylate Ser293 and Ser300, but only PDK1 is known to phosphorylate Ser232 [28]. Since no increase was observed in the phosphorylation of Ser232 in our cells, it is most likely that PDK3 is the main kinase affecting the phosphorylation, and therefore activity, of PDH in our model. A reduced PDH fractional flux, as observed in our mutant IDH1 cells, would facilitate the accumulation of metabolic building blocks required for cellular proliferation [12], [14]. However, our findings with regard to PDH are not entirely consistent with the clinical data, in which significant but opposite changes were observed in PDK1 and PDK3 expression precluding any conclusion with regard to PDH flux in human tumors.

In summary, we first performed an in-depth study in an immortalized astrocyte cell model that expresses heterozygous mutant IDH1 or wild-type IDH1. The IDH1 mutation in low-grade gliomas is likely an early event in tumorigenesis [4] and subsequent mutations follow, creating the characteristic cellular phenotype and natural history of disease in patients [1], [3]. Accordingly, our model might not fully reproduce the metabolic alterations associated with the human disease. However, because it provides a controlled system to isolate the effects of the IDH1 mutation, we hypothesized that this approach was valuable in identifying the metabolic events that result specifically from the IDH1 mutation. More importantly, by comparing with TCGA data, this led us to identify a metabolic change that also appears to be relevant in the clinical setting. To the best of our knowledge this is the first study showing that presence of the IDH1 mutation leads to modulation of PC flux in glioma cells. Combined with the clinical data, our findings indicate that increased PC expression and activity could be essential for survival of mutant IDH1 cells and, as such, could serve as a potential therapeutic target for mutant IDH1 gliomas either alone or in combination with other therapies.
